# Prescribing knowledge and skills of final year medical students in Nigeria

**DOI:** 10.4103/0253-7613.45150

**Published:** 2008

**Authors:** K.A. Oshikoya, J.A. Bello, E.O. Ayorinde

**Affiliations:** 1 Department of Pharmacology, Lagos State University College of Medicine, Ikeja, Lagos, Nigeria; 2 Department of Paediatrics, Lagos State University Teaching Hospital, Ikeja, Lagos, Nigeria

**Keywords:** Knowledge, medical student, prescribing, skill

## Abstract

**Objectives:**

To assess the knowledge of final year medical students in Nigeria, about good prescribing and the application of this knowledge to their prescribing skills.

**Materials and Methods:**

Thirty four final year medical students of the Lagos State University College of Medicine (LASUCOM), Ikeja, were interviewed with a structured questionnaire that assessed their knowledge on the principles of good prescribing. They were also requested to write a prescription, based on a paediatric clinical scenario of malaria and upper respiratory tract infection. The prescription was used to assess their prescribing skills.

**Results:**

Thirty one (91.18%) students knew that rational prescribing involved prescribing correct dosage of an appropriate medicine formulation. Factors considered important by the students to prescribe rationally were: Potential benefit: risk ratio of a medicine - 33 (97.06%); good knowledge of pharmacology - 29 (85.29%) and pathophysiology of the disease to be treated - 24 (70.59%); and safety of an alternative medicine to be used - 24 (70.59%). An average of 3.71 medicines was prescribed for a child suspected to have malaria. Antimalarials (38.24%) and paracetamol (20%) were the most frequently prescribed medicines. The name and signature of the prescriber were available in 51.61% and 58.06% prescriptions, respectively. Less than 50% prescriptions had the name, case file number, age and gender of the patient.

**Conclusion:**

The final year medical students of LASUCOM would require theoretical and practical teaching of principles of rational prescribing to improve their prescribing knowledge and skills.

## Introduction

Evidence of poor prescribing is abundant in Nigeria.[1] Antimalarials and antibiotics are prescribed for children, with little regard for resistance and adverse drug reaction development.[1,2] Prescription errors are very common,[3,4] especially with fresh doctors.[5] The basic problem which contributes to the irrational prescribing is that the medical students are not adequately instructed.[6] Clinical pharmacology and therapeutics is taught in only a few of the Nigerian medical schools. Where it is taught, the knowledge imparted is only theoretical. Specialists in this area are scarce in Nigeria, making the teaching and training in good prescribing inadequate. It has been suggested that all medicals schools should have an identified individual to coordinate the teaching of prescribing and therapeutics.[7] Such a trainer may not necessarily be a clinical pharmacologist. The house officers and interns are often left to fend for themselves during their training[8] and their prescriptions are hardly supervised. Thus, those who are ill-equipped in rational prescribing continue to make medication errors.

Modern medicines are too powerful an intervention for the newly qualified doctors to be allowed to prescribe without providing evidence of competence.[6] It has been reported in the UK that the root cause of prescribing errors among final year medical students is the lack of knowledge base that integrates scientific knowledge with clinical know-how.[9]

Prescribing is becoming increasingly difficult amongst doctors in Nigeria,[1,2] and the inherent risks of ADRs have increased.[10] Polypharmacy is increasing and the extremes of age (paediatric and the geriatric population) are at greater risk of ADRs with the modern medicines. Young doctors, therefore, need a firm grounding in the principles of clinical pharmacology, linked to practical therapeutics,[11] so that they can weigh the potential benefit and risk of treatment, understand the sources of variability in medicine response, base prescribing decisions on sound evidence, and monitor medicine effects appropriately.

This study, therefore, aims at assessing the knowledge of final year medical students in the Lagos State University College of Medicine, (LASUCOM), Ikeja, Nigeria, on the principles of good prescribing, and testing their prescribing skills. The result of this study can serve as a reference for future studies. We also aim at suggesting strategies for teaching practical-based rational prescribing based on the identified problems.

## Materials and Methods

The Lagos State University College of Medicine is a young medical school, established in 1998. The final year medical students that took part in this study were the second set of medical graduates from this college. The students were identified by their identity cards, during a revision class. Out of 37 students, 34 consented to participate in the study. They were interviewed with a structured questionnaire, which was filled on the spot. They were also requested to write a prescription based on a paediatric clinical scenario of malaria and upper respiratory tract infection, since these ailments are very common in Nigeria and other sub-Saharan African countries.[12,13] While the questionnaire assessed the knowledge of the students on principles of good prescribing, the prescription assessed their skills. Clearance was obtained from the provost of the college and the questionnaire was administered by all the researchers. The questionnaire was pretested at the College of Medicine University of Lagos (CMUL).

The results were grouped as follows: knowledge of good prescribing, prescribing skills, and application of prescribing knowledge to prescribing skill. Prescribing skills were analysed based on the students' quality of prescription, compliance with good prescription practice, and the WHO's core prescribing indicators. The data was analysed with SPSS version 13. Ranking and adherence to prescribing indicators were compared by Chi-square, at a significance level of *P*< 0.05.

## Results

### Knowledge of good prescribing

The 34 medical students who took part gave a response rate of 91.89%. Their mean age was 28.09 ± 2.24 years, with male: female ratio being 0.54: 1. A good number of the students were able to identify the components of rational prescribing. Thirty one (91.18%) students knew that rational prescribing involved prescribing the correct dosage of an appropriate medicine formulation; 23 (67.65%) knew that rational prescribing involved prescribing the appropriate medicine, specifying correct frequency of medication administration, and specifying the correct length of time of the medication prescribed. A reasonable number of the students knew the basis of rational medicine prescribing. Twenty nine (85.29%) based rational prescribing on the knowledge of pharmacology; 24 (70.59%) based it on a sound knowledge and understanding of the pathophysiology of the disease to be treated; 23 (67.65%) based it on the knowledge of the risks associated with the medicine; and, 21 (61.77%) based it on the knowledge of the benefits of the medicine. A majority of the students (97.06%) believed the potential benefit: risk ratio of a medicine should be determined, before it was prescribed. Many students (79.41%) believed that the benefit: risk ratio could be increased by a highly effective medicine with negligible ADRs and if such medicine was the only one available for use. Similarly, safety of alternative medicine (70.59%) to the prescribed medicine, efficacy of the alternative medicine (64.71%), seriousness of the problem to be treated (58.82%), and seriousness and frequency of possible ADRs of the medicine of choice (58.82%) were other factors considered by the students, to influence the benefit: risk ratio of a medicine. Only 16 (47.06%) believed that life threatening diseases could enhance benefit: risk ratio of a medicine.

All the students were able to define evidence-based medicine as the practice of medicine that based clinical decision to treat a patient on the best scientific evidence at the time of treating a disease. Twenty seven (79.41%) students correctly identified systematic review of clinical trials, intellectual searching and analysis of both published and unpublished data that are made available in databases as the source of evidence-based medicine. Other sources identified were clinical meetings and presentations (67.65%), review articles (64.71%), and talking to doctors or listening to their lectures (47.06%). An average of 24 (70.59%) students were able to rank high WHO's core prescribing indicators [low number of medicines prescribed per prescription, high percentage of generic prescription, low percentage of antibiotic prescription, low percentage of prescribed injectables, and high percentage of medicines prescribed from essential medicine list (EML)]. Of these core indicators, low percentage of antibiotics prescription was ranked the highest and low percentage of injectables prescription ranked the lowest. Overall, prescription based on parental influence (73.53%) and high rate of prescription by brand names (73.53%) were ranked the lowest of the listed prescribing quality indicators.

The students would prescribe oral rehydration solution 27 (79.41%), antibiotics 23 (67.65%), multivitamins 16 (47.06%), ascorbic acid 15 (44.12%), blood tonic 7 (20.59%), antimalarials 6 (17.65%) and antidiarrheal medicine 6 (17.65%) to a child with bacillary dysentery and upper respiratory tract infections.

Twenty eight (82.35%) students agreed to paediatric prescription in dosage per weight; 24 (70.59%) agreed to dosages based on a child's age; and 15 (44.12%) students respectively agreed to dosages in children based on their body surface area and the affordability of the medicine. Only three (8.82%) agreed to paediatric medicine prescription based on their height. Twenty (58.82%) students agreed to syrup prescription in children in milligram (mg) per body weight, 16 (47.06%) agreed to syrup prescription in millilitres (ml), 14 (41.18%) agreed to tablet prescription to older children in mg per body weight, and, two (5.88%) agreed to tablet prescription in a unit number per dose. Twenty seven (79.41%) students believed that medicine dosages should be modified in some disease conditions. Only one student believed that dosage modification was unnecessary. Twenty eight (82.35%) students would prescribe antibiotics, if indicated, for a minimum of five days.

The students would prescribe injections when a patient was unconscious (24, 70.59%), vomiting (23, 67.65%), having diarrhoea (16, 47.06%); on request by the patient (15, 44.12%), and having poor appetite (14, 41.18%), fever (13, 38.24%) and lethargy (13, 38.24%).

### Prescribing skills

Only 31 of the 34 students that responded filled the prescription form. A total of 115 medicines were prescribed and paracetamol was the most prescribed medicine (n = 23; 20.0%). A total of 44 (38.24%) antimalarials were prescribed and artemesinin (n = 12; 10.40%) was the most prescribed antimalarial drug [Table 1]. The number of medicines prescribed ranged between 1 and 7;. Nineteen (61.29%) of the prescriptions had three or less medicines. The average number of medicines prescribed per patient was 3.71.

**Table 1:** List of medicines

*Medicines'name*	*Number of times prescribe (n = 115)*	*Percentage of total*
Antipyretic		
Paracetamol	23	20.00
Antimalarials		
Artemesinin	12	10.43
Artemether + lumefantrine	11	9.57
Amodiaquine	8	6.95
Chloroquine	8	6.95
Sulphadoxime + Pyrimethamine	4	3.48
Artemesinin + Amodiaquine	1	0.86
Antibiotics		
Amoxicillin + Clavulanic acid	4	3.48
Amoxicillin	2	1.74
Ciprofloxacin	2	1.74
Metronidazole	2	1.74
Haematinics		
Vitamin B complex	6	5.22
Ascorbic acid	4	3.48
Multivitamins	4	3.48
Folic acid	2	1.74
Blood tonic	2	1.74
Antihistamines		
Metoclopramide	2	1.74
Chlorpheniramine	2	1.74
Promethazine	2	1.74
Others		
Oral rehydration solution	10	8.70
Cough mixture	2	1.74
Intravenous fluid	2	1.74

The prescriber's name and signature were available in 51.61% and 58.06% prescriptions, respectively. The patient's name, age and gender, respectively were available in 15 (43.39%) prescriptions. Only 13 (41.93%) prescriptions had the case file number.

Table 2 shows the analysis of the prescriptions with regard to date, name of medicine, strength, dose unit, instruction to patients, and legibility of the prescription. The date of prescription was provided in 14 (45.16%) prescriptions. All the medicines were legibly prescribed. The generic name of medicines was used in 15 (48.39%) prescriptions. No prescription was written in only brand name, while both acronym and generic name were provided in 16 (51.61%). The core prescribing indicators from the medical students' prescriptions and the paediatric outpatient prescriptions of the affiliate teaching hospital (LASUTH) have been compared in Table 3. There was no significant difference in the average number of medicines prescribed per prescription, medicines prescribed by generic name, and medicines prescribed from EML. The scoring parameters for appropriate ranking of the prescribing indicators is summarised in Table 4. Ranking of any of the WHO core prescribing indicators as first to fifth was taken as a “yes” response, while it was taken as “no” response if the ranking was beyond fifth.

**Table 2:** Further analysis of information present on the respondents' prescriptions

*Parameter*	*Number from prescription (n = 31)*	*Percentage of total prescription*
Date of prescription		
Provided	14	45.16
Medicine names		
Generic only	15	48.39
Acronym	13	41.93
Mixed	16	51. 61
Readable	31	100.00
Strength of medications		
Included for all medicines	14	45.16
Included for some medicines	9	29.03
Not included for all medicines	8	25.81
Expressed in ml and unit tablet	23	74.19
Dose units		
Included for all medicines	11	35.48
Included for some medicines	11	35.48
Not included for all medicines	9	29.03
Instructions for patient use		
Included for all medicines	0	00.00
Included for some medicines or partial instructions	0	00.00
Missing for all medicines	31	100.00
Prescriber handwriting		
Legible	31	100.00

**Table 3:** Comparing the core prescribing indicator values from the respondents' prescriptions and the paediatric outpatient prescriptions of the affiliate teaching hospital (LASUTH)

*Parameter*	*LASUCOM*	*LASUTH^1^*
Average number of medicines prescribed per prescription	3.71	3.70
Medicines prescribed by generic name (%)	58.06	7.30
Antibiotics prescribed (%)	8.69	41.40
Injections prescribed (%)	16.52	18.00
Medicines prescribed from essential list (%)	79.13	84.80

**Table 4:** Scoring parameters for appropriate ranking of the prescribing indicators

*Prescribing indicators*
WHO core prescribing indicators
Low percentage of antibiotic prescription
Low number of medicines prescribed per prescription
High percentage of generic prescription
High percentage of medicines prescribed from essential list
Low percentage of prescribed injectables
Other indicators
Low prescription of nutritional supplement vitamins and iron tonic
Low rate of prescribing of compound analgesics relative to paracetamol
High rate of prescribing cheap medicines
Prescribing based on parental influence
Prescribing newly promoted medicines
Prescription by brand and other names

Ranking of any of 1-5 as 1^st^ to 5^th^ is taken as a “yes” response. Ranking of any of 6-7 as 6^th^ or 7^th^ is taken as a “yes” response. Ranking of any of 8-11 as 8^th^ to 11^th^ is taken as a “yes” response.

### Application of knowledge of good prescribing to prescribing skill

The ranking of the prescribing indicators by the students was compared with their adherence in prescription writing in Table 5. It is clear that some of the indicators, including the highly ranked WHO's core prescribing indicators, were not strictly adhered to. There were significant differences in their ranking of the prescribing indicators and the prescriptions written.

**Table 5:** Comparing respondents' knowledge of prescribing indicators with its adherence to prescription writing

*Order of ranking*	*Ranking of prescribing indicators in percentage*	*Adherence to prescribing indicators in percentage*	*P- value*
High ranking			
Low percentage of antibiotic prescription	87.10	8.69	< 0.0001
Low number of medicines prescribed per prescription	83.87	61.29	< 0.001
High percentage of generic prescription	80.65	58.06	< 0.001
High percentage of medicines prescribed from essential list	80.65	79.13	0.837
Low percentage of prescribed injectables	58.06	16.52	< 0.0001
Low prescription of nutritional supplement vitamins and iron tonic	45.16	15.66	< 0.0001
Low ranking			
Prescribing newly promoted medicines	70.97	*21.74	< 0.0001
Prescription by brand and other names	80.65	51.61	< 0.001

* ACT= Artemesinin Combined Therapy and *ciprofloxacin (newly promoted medicines in Nigeria).

## Discussion

The result of this study shows that knowledge of good prescribing by the final year medical students is deficient. A majority of the students were able to correctly define rational prescribing.[14] Principles of good prescribing are based on sound knowledge and understanding of the pathophysiology of the disease to be treated, and the knowledge of risks and benefits of the medicine.[14,15] These principles were well identified by most of the students. Life threatening diseases have been reported as some of the conditions that could enhance benefit: risk ratio of a medicine.[14] Unfortunately, only 47.06% students were able to identify this. Rational prescribing can be achieved by practising evidence-based medicine. Even though this is not fully practised in Nigeria,[1,2] the awareness of 79.41% students that evidence-based medicine can be obtained from systematic review of clinical trials, intellectual searching, and analysis of both published and unpublished data that are made available in databases is an encouragement that rational prescribing is achievable in Nigeria. The ability of 70.59% students to rank WHO's core prescribing indicator values[1] high is commendable. It shows their tendency to comply with the WHO guidelines for prescribing. Parental influence on doctors to prescribe medicines, even when not necessary, has been reported in the UK[16] and this is not uncommon in Nigeria.[1] Ranking prescription based on parental influence and brand names as low, by 73.53% students, is praiseworthy and supports the possibility of achieving rational prescribing.

Bacillary dysentery and upper respiratory tract infections in children being self limiting, do not require antibiotics.[1,17] However, 67.65% students [Table 1] showed an inclination for prescribing antibiotics for these conditions, thereby causing antibiotic abuse and thus promoting resistance. This result is also a reflection of the inadequate knowledge of the pathophysiology of the diseases by the students.

Although dosage schedules for children have been determined by clinical trial or experience, it is often scaled down from adult dosage using bodyweight, body-surface area, age or by a combination of these parameters.[18,19] Each of these methods has its own advantages and disadvantages.[18] Only 82.35% students agreed to prescribe to children based on their body weight, while 70.59% were ready to prescribe based on their age. This result, however, does not correlate with the percentage of students that would prescribe both syrup and tablet in milligram. Rather, syrup prescription in millilitre and tablet in a unit number would be practised by many of the students. We have earlier reported dosage errors associated with prescription in children in millilitres and unit tablet number.[3] The fact that 47.06%, 44.12% and 41.18% students would prescribe injections for patients with diarrhoea, on request and poor appetite, respectively, is a matter of concern. This might have resulted from lack of knowledge about the hazards of injections.[1]

In prescribing skills, the prescriptions were found to be deficient. Malaria was the provisional diagnosis for the hypothetical case presented for prescription writing, which explained the high antimalarial prescription. Even though malaria resistance had been reported in Nigeria,[3] necessitating the use of artemesinin and its derivatives as first line medicines, these and the prescribed antibiotics (ciprofloxacin), as presented in Table 1, are too potent for the newly qualified graduates to prescribe without providing evidence of competence.[6]

Our findings of 51.61% prescriptions having the prescriber's name and 58.60% having the prescriber's signature, shows deficient prescribing. These results are lower than those reported in the affiliated teaching hospital (LASUTH).[1] These elements, according to the WHO, are essential when filling a prescription form.[20] Besides, they are very useful to the dispensing pharmacist for contacting the prescriber in case of any clarification.

Concerning patient information, 43.39% prescriptions had the patient name and age. These elements are essential to be filled on a prescription form.[21] Besides, patients' name and address are useful in tracing a patient, in case of prescribing and dispensing errors, while the age is useful at estimating the patient's weight when unconscious or uncooperative.[20]

The extent of acronyms (41.93%), generic names (48.39%), and a mixture of acronym, brand and generic names (51.61%) in the prescriptions of the students is similar to what had been reported earlier.[1] Using generic names in prescriptions gives flexibility to medicine stocking and to the dispensing pharmacist; besides, it is economical.[1,22] However, the use of brand names may be acceptable when problems of bioavailability are expected.[1,23]

We also found that 29.03% of the prescriptions did not include the strength of the medication; dose units were not included in 29.03% and all the prescriptions were deficient in instructions for the patient on how to use the medicines. Apparently, these parameters might have been left to the pharmacist to decide upon and the implication for the duration of therapy would have been dependent on the individual pharmacist. The strength of medication is particularly needed when the pharmaceutical product is available in different strengths.

An average prescription rate per patient of 3.71 obtained from this study was similar to LASUTH[1] [Table 3] and other studies from Nigeria.[2,24] The core prescribing indicator values from the medical students' prescriptions was similar to LASUTH, except for the differences in their generic prescriptions (58.06% from LASUCOM and 7.30% from LASUTH) and antibiotics prescriptions (8.69% from LASUCOM and 41.40% from LASUTH). These findings further support the fact that both theoretical and practical teaching of prescription writing could achieve good and rational prescribing.[25]

The knowledge of the students did not match their prescribing skills. Low prescription rate per patient was ranked high, but 3.81 was obtained from this study. This value is >3 recommended by WHO.[21] Generic prescription was also ranked high, but only 58.06% was obtainable [Table 5]. These results, therefore, emphasize the need for practical teaching of prescription writing.

## Conclusion

The LASUCOM final year medical students are deficient in knowledge of good prescribing and lack the skills required for rational prescribing. Theoretical and practical teaching of the principles of good prescribing would be necessary to improve their prescribing knowledge and skills. Also, the prescribing knowledge and skills of the students should be assessed regularly.
